# Trends in Out-of-Pocket Cost of Glucagon, 2010-2020

**DOI:** 10.1001/jamanetworkopen.2022.29428

**Published:** 2022-08-30

**Authors:** Margaret Zupa, Robert Feldman, Jing Luo

**Affiliations:** 1 Division of Endocrinology and Metabolism, University of Pittsburgh School of Medicine, Pittsburgh, Pennsylvania; 2 Center for Research on Health Care Data Center, University of Pittsburgh, Pittsburgh, Pennsylvania; 3 Division of General Internal Medicine, University of Pittsburgh School of Medicine, Pittsburgh, Pennsylvania

## Abstract

This cross-sectional study investigates trends in out-of-pocket costs for unmixed and novel glucagon formulations among patients with Medicare Advantage and commercial insurance from 2010 to 2020.

## Introduction

Severe hypoglycemia results in 242 000 emergency department visits annually.^1^ More than 20% of patients with type 2 diabetes and all patients with type 1 diabetes in the United States are at risk of severe hypoglycemia due to insulin or sulfonylurea use.^2^ Glucagon is a life-saving medication for emergency treatment of severe hypoglycemia that can be administered outside of health care settings, where most episodes occur.

The American Diabetes Association guidelines recommend that patients at risk of moderate to severe hypoglycemia have access to glucagon.^3^ This is especially important for young children, who are at increased risk owing to their inability to communicate symptoms.^4^ However, an analysis of nationally representative claims data found that 3.5% of adults with type 1 diabetes and 8% of adults with type 2 diabetes who had prior severe hypoglycemia filled a glucagon prescription in 2014.^5^ Studies of insulin and other diabetes medications found that high out-of-pocket costs (OOPCs) were associated with low rates of use.^6^ In this study, we sought to assess trends in the OOPCs of unmixed and novel glucagon formulations.

## Methods

The University of Pittsburgh Institutional Review Board determined that this cross-sectional study was exempt from review and informed consent because it used deidentified claims data and was not human participants research. This study followed the Strengthening the Reporting of Observational Studies in Epidemiology (STROBE) reporting guideline. We assessed trends in wholesale acquisition cost (WAC), an estimate of the manufacturer’s list price, for glucagon formulations over time using the AnalySource drug pricing database. Next, we examined trends in OOPCs paid by patients using the Optum deidentified Clinformatics Data Mart, which includes administrative claims from large commercial and Medicare Advantage (MA) health plans. Analyzed claims included any formulation of glucagon from January 1, 2010, through September 30, 2020. Given that there were only 24 prescription fills of novel premixed liquid glucagon injection, it was excluded from analysis (eFigure in the Supplement). Analysis was performed using R statistical software version 4.1.2 (R Project for Statistical Computing) from November to December 2021.

## Results

The WAC of unmixed glucagon increased 192% from 2010 to 2020 (Figure). The median OOPC of unmixed glucagon was less than $37.00 for commercially insured beneficiaries and was $0 for MA beneficiaries throughout the study period (Table). Costs increased for MA beneficiaries in the 75th percentile of OOPC, from $25.00 to $40.00 from 2010 to 2020. The 2020 median OOPC of novel intranasal glucagon, which was introduced in 2019, was $15.00 for commercially insured beneficiaries and $10.00 for MA beneficiaries.

**Figure.**
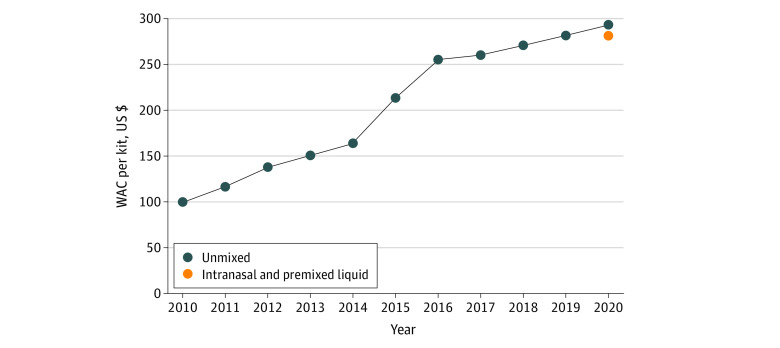
Wholesale Acquisition Cost (WAC) of Glucagon Premixed liquid glucagon prefilled syringe, autoinjector, and intranasal glucagon are represented as a single point given that the WAC for all 3 formulations was $280.80. WAC is per single-use kit.

**Table.** Volume of Prescription Fills and OOPCs for Unmixed and Intranasal Glucagon

Year	Prescription fills, No.	OOPC, median (IQR), $^a^	
		Commercial insurance^b^	Medicare Advantage
**Unmixed glucagon**			
2010	15 691	27.04 (14.88-37.45)	0 (0-25.00)
2011	16 946	29.29 (17.57-41.00)	0 (0-21.59)
2012	17 961	34.14 (22.76-39.83)	0 (0-25.61)
2013	19 531	33.61 (22.40-39.21)	0 (0-24.65)
2014	18 943	33.09 (16.54-38.60)	0 (0-24.81)
2015	19 925	33.11 (5.52-41.39)	0 (0-33.11)
2016	22 632	32.67 (0-43.55)	0 (0-32.67)
2017	24 303	31.87 (3.25-42.49)	0 (0-37.18)
2018	25 479	36.43 (0-41.63)	0 (0-36.43)
2019	24 906	35.87 (0-44.84)	0 (0-25.62)
2020^c^	14 748	30 (15.00-45.00)	0 (0-40.00)
**Intranasal glucagon**			
2019	449	10.89 (0-32.03)	7.71 (0-15.54)
2020^c^	6061	15.00 (7.50-35.00)	10.00 (0.98-23.50)

Abbreviation: OOPC, out-of-pocket cost.

^a^ Defined as the sum of copays and deductibles; costs were adjusted for inflation based on January 2020 dollars and scaled per single-use kit.

^b^ Insurance type was based on the primary payer at the time of the glucagon prescription fill.

^c^ Through September 30, 2020.

## Discussion

This cross-sectional study found that WAC rose steadily, while the median OOPC for unmixed glucagon was stable for commercially insured patients and $0 for most MA beneficiaries from 2010 to 2020. The difference in OOPCs between commercial and MA beneficiaries may be associated with incentives for MA plans to lower total costs, such as those potentially resulting from hypoglycemia-related emergency department visits. Alternatively, MA beneficiaries may wait to fill glucagon prescriptions until they reach catastrophic coverage, potentially suggesting that these patients lack access to life-saving medications earlier in the year. However, the median OOPC of unmixed glucagon increased for MA beneficiaries with the highest OOPCs (4th quartile). There are multiple possible reasons that may underly this trend, including MA plans passing on higher WAC to beneficiaries or beneficiaries choosing plans with more cost sharing over time.

Our study has several limitations. Results may not generalize to patients who are insured through traditional Medicare or Medicaid or are uninsured. In addition, we were unable to assess instances in which high OOPCs prevented patients from filling the prescription (primary nonadherence) or the association of manufacturer patient-assistance programs and coupons with OOPCs for commercial beneficiaries.

Cost-sharing should not serve as a major barrier to accessing glucagon for MA and commercially insured patients. Further work should assess other barriers to guideline-concordant glucagon uptake among patients at risk of severe hypoglycemia. Such barriers may include awareness among patients and primary care, emergency, and diabetes clinicians of the availability of this potentially life-saving treatment. In addition, future studies may examine the association of novel glucagon formulations, which had similar OOPCs to those of unmixed glucagon and may be easier to administer, with uptake in the coming years.

## Notes

Supplement. **eFigure.** Flow Diagram of Glucagon Claims Selection With Exclusion
