# Supplementary material for: Trends in Out-of-Pocket Cost of Glucagon, 2010-2020
Source: JAMA Netw Open. 2022 Aug 30;5(8):e2229428. doi: 10.1001/jamanetworkopen.2022.29428 (PMC9428735; doi:10.1001/jamanetworkopen.2022.29428)
Supplement: Supplement. — eFigure. Flow Diagram of Glucagon Claims Selection With Exclusion [file jamanetwopen-e2229428-s001.pdf]

Supplemental Online Content

Zupa M, Feldman R, Luo J. Trends in out-of-pocket cost of glucagon, 2010-2020. *JAMA Netw Open*. 2022;5(8):e2229428. doi:10.1001/jamanetworkopen.2022.29428

eFigure. Flow Diagram of Glucagon Claims Selection With Exclusion

This supplemental material has been provided by the authors to give readers additional information about their work.

© 2022 Zupa M et al. *JAMA Network Open.*

eFigure. Flow Diagram of Glucagon Claims Selection With Exclusion

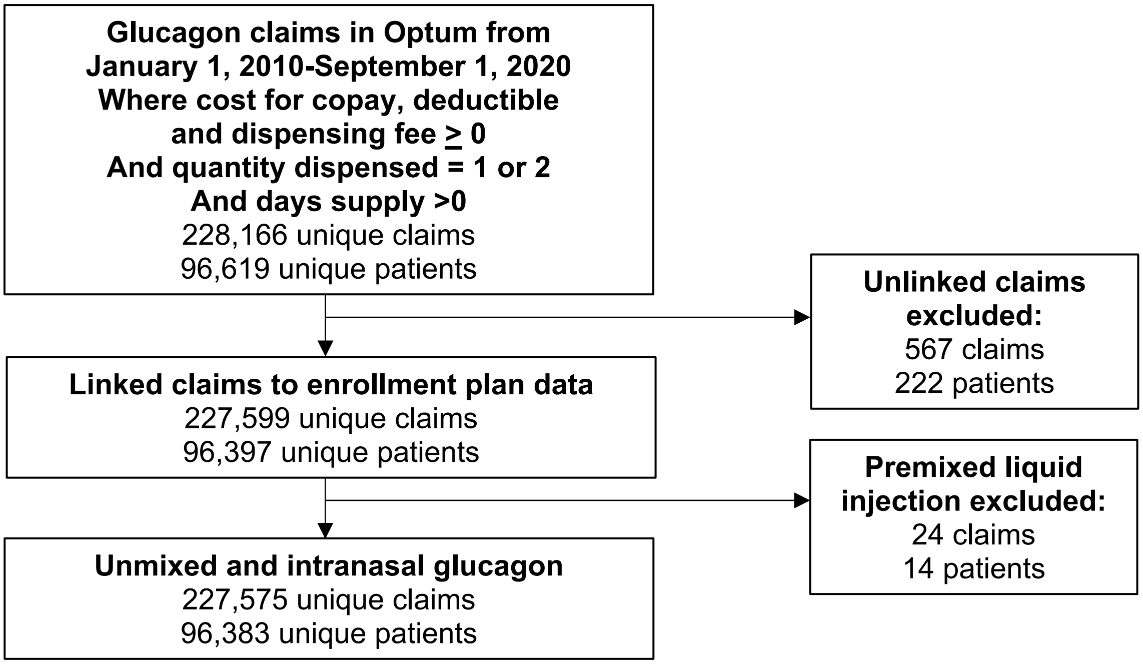

© 2022 Zupa M et al. *JAMA Network Open.*
